# Evaluation of a Web-Based Intervention for Multiple Health Behavior Changes in Patients With Coronary Heart Disease in Home-Based Rehabilitation: Pilot Randomized Controlled Trial

**DOI:** 10.2196/12052

**Published:** 2018-11-19

**Authors:** Yan Ping Duan, Wei Liang, Lan Guo, Julian Wienert, Gang Yan Si, Sonia Lippke

**Affiliations:** 1 Department of Sport and Physical Education Faculty of Social Sciences Hong Kong Baptist University Hong Kong China (Hong Kong); 2 Cardiac Rehabilitation Centre Guangdong General Hospital Guangzhou China; 3 Department of Psychology & Methods Jacobs University Bremen Bremen Germany; 4 Sport Psychology Centre Hong Kong Sports Institute Hong Kong China (Hong Kong)

**Keywords:** eHealth, physical activity, diet, cardiac rehabilitation, health resources

## Abstract

**Background:**

Web-based and theory-based interventions for multiple health behaviors appears to be a promising approach with respect to the adoption and maintenance of a healthy lifestyle in cardiac patients who have been discharged from the hospital. Until now, no randomized controlled trials have tested this assumption among Chinese rehabilitation patients with coronary heart disease using a Web-based intervention.

**Objective:**

The study aim was to evaluate the effect of an 8-week Web-based intervention in terms of physical activity (PA), fruit and vegetable consumption (FVC), lifestyle changes, social-cognitive outcomes, and health outcomes compared with a waiting control group in Chinese cardiac patients. The intervention content was theory-based on the health action process approach. Self-reported data were evaluated, including PA, FVC, healthy lifestyle (the synthesis of PA and FVC), internal resources (combination of intention, self-efficacy, and planning), and an external resource (social support) of PA and FVC behaviors, as well as perceived health outcomes (body mass index, quality of life, and depression).

**Methods:**

In a randomized controlled trial, 136 outpatients with coronary heart disease from the cardiac rehabilitation center of a hospital in China were recruited. After randomization and exclusion of unsuitable participants, 114 patients were assigned to 1 of the 2 groups: (1) the intervention group: first 4 weeks on PA and subsequent 4 weeks on FVC and (2) the waiting control group. A total of 2 Web-based assessments were conducted, including 1 at the beginning of the intervention (T1, N=114), and 1 at the end of the 8-week intervention (T2, N=83). The enrollment and follow-up took place from December 2015 to May 2016.

**Results:**

The Web-based intervention outperformed the control condition for PA, FVC, internal resources of PA and FVC, and an external resource of FVC, with an eta-squared effect size ranging from 0.06 to 0.43. Furthermore, the intervention effect was seen in the improvement of quality of life (*F*_1,79_=16.36, *P*<.001, η^2^=.17). When predicting a healthy lifestyle at follow-up, baseline lifestyle (odds ratio, OR 145.60, 95% CI 11.24-1886; *P*<.001) and the intervention (OR 21.32, 95% CI 2.40-189.20; *P*=.006) were found to be significant predictors. Internal resources for FVC mediated the effect of the intervention on the adoption of a healthy lifestyle (*R*^2^_adj_=.29; *P*=.001), indicating that if the intervention increased the internal resource of behavior, the adoption of a healthy lifestyle was more likely.

**Conclusions:**

Patients’ psychological resources such as motivation, self-efficacy, planning, and social support as well as lifestyle can be improved by a Web-based intervention that focuses on both PA and FVC. Such an intervention enriches extended rehabilitation approaches for cardiac patients to be active and remain healthy in daily life after hospital discharge.

**Trial Registration:**

ClinicalTrials.gov NCT01909349; https://clinicaltrials.gov/ct2/show/NCT01909349 (Archived by WebCite at http://www.webcitation.org/6pHV1A0G1)

## Introduction

### Background

Cardiovascular disease (CVD) is the leading cause of death worldwide, with coronary heart disease (CHD) being the major component [1]. In China, CVD was the number one cause of death in 2017, and CHD was the dominant component responsible for 51.6% of all CVD deaths [2]. Cardiac rehabilitation is widely recognized as an effective intervention to avoid further progression and relapse of CVD [3-5]. CHD patients receive the guidance provided on healthy lifestyle changes regarding physical activity (PA) and a healthy diet during rehabilitation in the hospital [6,7]. However, several studies have revealed that it is often difficult for patients to integrate and transfer these recommendations and learning outcomes into their daily life after discharge from the hospital [8,9]. Thus, they need internal and external resources, which can be supported by an extended rehabilitation aftercare when they are at home [10].

With the prevalence of internet use, Web-based interventions are widely developed and applied in the field of cardiac rehabilitation [11-13]. The effectiveness of Web-based interventions on PA and fruit and vegetable consumption (FVC) has been proven to facilitate healthy lifestyle changes after discharge among cardiac patients and people with cardiovascular risk profiles in the Western Hemisphere [14-16]. However, in the Eastern Hemisphere, and especially in China, a large body of Web-based rehabilitation interventions only focus on knowledge, education, and learning. Very few integrate individualized and comprehensive interventions that include educational, cognitive, and psychological elements [17,18].

This study applied the Health Action Process Approach (HAPA) [8] as the theoretical backdrop, which suggests that there are 2 distinctive phases during the health behavior change process. The first phase is the *motivation phase*, which plays an important role before a goal is set. Subsequently, individuals enter the *volition phase*, which plays an important role in planning and maintaining health behavior changes. This distinction allows interventions to be tailored based on variables that correspond to either the motivation or the volition phase. In particular, motivation must be built up before individuals can actually change unhealthy behaviors (eg, physical inactivity). Therefore, individuals with an unhealthy lifestyle and no intention to change it might benefit most from interventions that will increase risk perception, self-efficacy, and positive outcome expectancies, to support the formation of goal intentions that focus on healthy lifestyle changes [19]. The purpose is to lead toward a specific intention (eg, “I intend to eat five portions of fruit and vegetables today”). Once the intention is formed and the goal is set, individuals enter the volition phase.

In the volition phase, people benefit most from planning interventions, which can help cross the so-called intention behavior gap [14]. In this phase, individuals learn how to make specific plans (eg, when, where, and how to eat fruit and vegetables), determine priorities, and translate their action plans into behavior. Such self-regulatory planning skills are necessary to maintain progress once people initiate a healthy behavior. Positive behavior change will be guided by self-efficacy, as self-efficacy regulates how much effort is invested in goal achievement, and how much persistence is maintained if obstacles and setbacks occur. In addition, promoting behavior-specific social support from people’s social context is equally important in preventing relapse [8]. In general, cardiac rehabilitation patients need internal (forming and improving intention, self-efficacy, and planning) and external (forming behavior-specific social support) resources to support them to adopt and maintain a healthy lifestyle after their discharge from the hospital.

Until now, how internal and external resources interplay with lifestyle change in cardiac patients (eg, following recommendations for PA and FVC) has not been fully addressed. Interventions that integrate both internal and external resources may enhance social cognitions, which in turn can lead to the adoption of a healthy lifestyle. Thus, mediation analysis might disclose the underlying mechanisms of such an intervention. All of this has been researched in the Western Hemisphere [15,20] but not in the Eastern Hemisphere, and therefore, the purpose of this study is to fill this gap.

### Aim and Hypotheses

The main research aim of this study was to examine the efficacy of an 8-week Web-based intervention compared with a waiting control group (WCG) to improve PA and FVC in Chinese rehabilitation patients with CHD after hospital discharge. Effects on single health behaviors, healthy lifestyle (the synthesis of PA and FVC), social-cognitive indicators for PA and FVC (internal and external resources), and perceived health outcomes (body mass index [BMI], quality of life, and depression) were tested (see hypotheses 1 to 3). The second aim of this study was to examine whether changes in internal resources (combination of intention, self-efficacy, and planning) and an external resource (social support) mediate the effect of the intervention on changes in lifestyle (hypothesis 4).

The main intervention effects were hypothesized in terms of (1) self-reported increase in PA and FVC behavior (single behavior indicators; hypothesis 1a) and adoption of a healthy lifestyle (hypothesis 1b); (2) more improvements in indicators of internal resources (combination of intention, self-efficacy, and planning) and an indicator of external resources (social support; hypothesis 2); (3) improvement in health outcomes (BMI, quality of life, and depression level; hypothesis 3); and (4) the expectation that those patients who had increased internal resources (combination of intention, self-efficacy, and planning) and an external resource (social support) because of the intervention would be more likely to adopt a healthy lifestyle (mediation effect; hypothesis 4).

## Methods

### Study Design, Procedure, and Participants

This study was a pilot randomized controlled trial involving 1 intervention group (IG) and 1 WCG. There were 2 assessments: a pretest after randomization and before intervention start or the waiting time (T1, n=114) and a posttest at 8 weeks after the pretest (T2, n=83). The WCG was allowed to access the 8-week Web-based intervention after T2, once the IG had finished the intervention. All patients were informed about the purpose of the study with an informed consent form. The study received ethical approval by the Committee for the Use of Human & Animal Subjects in Teaching & Research of Hong Kong Baptist University (FRG1/12-13/064) and was registered with ClinicalTrials.gov (NCT01909349; Multimedia Appendix 1 [12]).

Enrollment and follow-up took place from December 2015 to May 2016. Outpatients with CHD were recruited face-to-face by the physician of the research team, with the assistance of a nurse in the cardiac rehabilitation center of a hospital in south China. The inclusion criteria were as follows: aged between 18 and 75 years, no restriction of physical mobility under the cardiac function at entry, no restriction of other relevant diseases such as diabetes or fruit allergies, sufficient reading and writing skills in Chinese, internet access via a computer at home, and mobile access.

**Figure 1:**
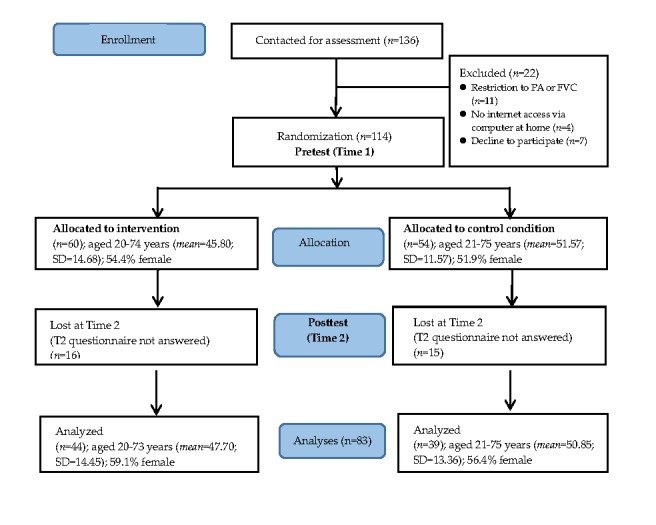
Flowchart of participant progress throughout the study phases. FVC: fruit and vegetable consumption; PA: physical activity.

Figure 1 shows the flowchart of patients from enrollment in the study to allocation to the 2 groups (IG and WCG) and posttest after 8 weeks. A total of 136 patients with CHD were recruited. Of these, 16.2% (22/126) were excluded after random allocation due to restrictions in terms of PA or FVC (n=11), no internet access via a computer at home (n=4), or because they declined to participate (n=7). Subsequently, 83.8% (114/136) eligible patients fulfilled the online registration and provided personal information within 1 week during the period of hospital rehabilitation. In total, 60 patients (52.6%) in the IG and 54 patients (47.4%) in the WCG were included. During the last week before they were discharged from the hospital, patients in both groups were invited to complete the first online questionnaire (pretest; T1). Patients in the IG were encouraged to access a Web-based health program via their home computer once a week during the following 8 weeks, whereas patients in the WCG did not receive any treatment. Upon completion of the 8-week intervention, patients in both groups were invited to fill in the second online questionnaire (posttest; T2). The final longitudinal sample consisted of 83 (72.8%) patients, including 44 (53%) in the IG, and 39 (47%) in the WCG (Figure 1).

All website links for the questionnaire surveys at T1 and T2 as well as for the weekly intervention program were delivered via email. To boost the engagement of patients, short message service (SMS) text messages were sent as reminders. Furthermore, the nurse contacted participants via phone calls once per week before each intervention session and at the 2 measurement points to remind the patients. Moreover, patients were offered a 60 renminbi (RMB; US $9) telephone recharge card as an incentive in exchange for their participation in the study and completion of the 2 data collection waves.

### Intervention

The 8-week Web-based intervention started with PA in the first 4 weeks, followed by the FVC in the later 4 weeks. This sequential design falls into the previous study results, suggesting PA might act as gateway behavior [21,22], and PA modules are the most favored modules in tailored electronic health (eHealth) lifestyle promotion [23].

The intervention content was designed based on the HAPA theory. Accordingly, social-cognitive variables of PA and FVC were targeted in the intervention (Multimedia Appendix 2). In particular, the targeted variables were as follows: week 1 and week 5—risk perception, outcome expectancies, and goal setting; week 2 and week 6—development of action plans; week 3 and week 7—revision and adjustment of previous action plans and development of coping plans; and week 4 and week 8—revision and adjustment of previous coping plans and development of behavior-specific social support. Self-efficacy was a fixed intervention variable that was included from week 1 to week 8. Multimedia Appendix 2 indicates intervention variables and content for each week.

In addition, selected behavior change techniques were addressed in the intervention to facilitate the implementation and maintenance of behavior [24]. For example, 2 types of feedback were provided: patients received individualized feedback on their self-reported behavior performance 4 weeks ago, 3 weeks ago, 2 weeks ago, and 1 week ago. Criterion-based feedback was also presented (eg, accumulated at least 150 min with moderate intensity of PA per week and 5 portions of FVC per day). The examples of the feedback can be found in a separate publication [25]. Moreover, examples of role models were provided throughout the intervention to support patients to set goals, develop plans, and increase their self-efficacy.

### Measures

All variables were self-reported online at pretest (T1) and posttest after the 8-week intervention period (T2).

#### Demographic Information

Sociodemographic information such as gender, age, level of education (low-level: primary school; high-level: high school, and university or college), and relationship status (single or in a relationship) was assessed. In addition, self-reported body height (cm) and weight (kg) were collected at T1 and T2.

#### Health Behavior (Single Behavior Indicators)

The level of PA was assessed using an adaption of the Chinese short version of the International Physical Activity Questionnaire [26]. Patients indicated how often per week and how long each time they performed vigorous PAs (eg, fast bicycling and intensive swimming) and moderate physical activities (eg, carrying light loads and bicycling at a regular pace) during the last week. The total PA score (in min/week) for each patient was obtained by summing up all questions.

FVC during the past 7 days was assessed with 4 items, including *raw vegetables*, *fruits*, *fruit or vegetable juice*, and *cooked or steamed vegetables* [27]. For each item, patients were asked to count the number of portions or glasses of liquid, fruit, and vegetables they consumed on average per day (11 options such as 0, 0.5, 1, 1.5, 2, 2.5..., 5, or above). The total consumed portion was the sum of each item.

#### Combined Healthy Lifestyle Indicator

To combine the 2 behaviors, PA and FVC behaviors were categorized according to whether or not the patients met the health recommendations [20]. After exploring the usefulness of different criteria, the thresholds of 150 min of PA with moderate intensity per week and 5 portions of fruit and vegetables were chosen. Both criteria were validated and have been shown to be effective in improving health [28,29]. At T1, 60% of patients (50/83 eligible patients participating in T1 and T2) did not perform 150 or more min of PA with moderate intensity per week. Moreover, 71% (59/83) of patients did not eat 5 or more portions of fruit and vegetables per day. When both behaviors were combined, 84% (70/83) participants met only 1 or none of these 2 behavioral criteria and were categorized as having an unhealthy lifestyle at T1. Overall, 15% (13/83) of the patients met both behavior recommendations and were categorized as having a healthy lifestyle.

At T2, 50% of patients (42/83 eligible patients participating in T1 and T2) did not perform 150 or more min of PA with moderate intensity per week. In addition, 60% (50/83) of patients did not eat 5 or more portions of fruit and vegetables per day. When both behaviors were combined, 73% (61/83) of participants met only 1 or none of these 2 behavioral criteria and were categorized as having an unhealthy lifestyle at T1. In total, 26% (22/83) of patients met both behavior recommendations and were categorized as having a healthy lifestyle.

#### Social-Cognitive Indicators as Internal Resources of Behavior Change

##### Intention

Intention concerning PA was measured with the stem “On 5 days a week for 30 min, I have the intention to perform...” followed by 3 items such as “...strenuous sports activities,” “...moderate PA,” and “...mild PA, like walking” (Cronbach alpha=.40) [30]. Intention concerning FVC was assessed by the stem “I intend to...” followed by 3 items such as “...eat at least 5 servings of fruits and vegetables each day,” “...eat fruit and vegetables at every meal,” and “drink at least one fruit or vegetable juice every day” (Cronbach alpha=.51) [30]. The answers were indicated on a 4-point scale ranging from 1 “not true” to 4 “exactly true.”

##### Self-Efficacy

Self-efficacy for PA was assessed with 5 items such as “I am certain that I can permanently be physically active for at least 5 days a week for 30 minutes each day” (Cronbach alpha=.89) [31]. Self-efficacy for FVC was assessed by 5 items such as “I am certain that I can eat 5 portions of fruits and vegetables a day, even if it is sometimes difficult” (Cronbach alpha=.95) [32]. The answers were given on a 5-point scale ranging from 1 “don’t agree at all” to 5 “totally agree.”

##### Planning

The planning indicator was categorized into *action planning* and *coping planning*. Action planning was assessed by the stem “For the next month I have carefully planned...” followed by 3 items for PA such as “...which PA I will pursue” (Cronbach alpha=.94) or followed by 3 items for FVC such as “...what I will eat” (Cronbach alpha=.91) [32,33]. Coping plans were measured by the stem “For the next month I have carefully planned...” followed by 3 items for PA such as “...What I can do in difficult situations to stick to my intentions” (Cronbach alpha=.90) or followed by 3 items for FVC such as “...How I can eat healthy, even if something happened” (Cronbach alpha=.92) [32,33]. The answers were given on a 5-point scale ranging from 1 “strongly disagree” to 5 “strongly agree.”

#### Social-Cognitive Indicator as External Resources of Behavior Change

##### Social Support

This indicator was measured with 3 items for PA (Cronbach alpha=.72) and with 3 items for FVC (Cronbach alpha=.87) such as “My partner helps me/my family helps me/my friends and acquaintances help me to stay physically active” or “My partner helps me/my family helps me/my friends and acquaintance help me to eat healthy.” Answers were given on a 4-point scale ranging from 1 “totally disagree” to 4 “totally agree” [34].

#### Health Outcomes

##### Body Mass Index

BMI was calculated using the formula “BMI=weight (kg)/height (m^2^).” Body weight and body height were reported by the study participants.

##### Quality of Life

This indicator was measured using the Hong Kong version of the World Health Organization’s Quality of Life questionnaire (Brief version) [35]. Respondents were first asked about their general quality of life by the item “How would you rate your quality of life?” Study participants were assessed with a 5-point scale ranging from 1 “very poor” to 5 “very good” and by 7 items on physical health subdomains. Such subdomains were “How satisfied are you with your ability to perform your daily living activities?” Response categories used a 5-point scale ranging from 1 “very dissatisfied” to 7 “very satisfied” (Cronbach alpha=.87).

##### Depression

Level of depression was measured using the Chinese translated Center for Epidemiological Studies-Depression (CES-D) scale. Respondents were asked with the stem “In the past week how often I feel...” followed by 20 items such as “...I was bothered by things that usually don't bother me.” Respondents were asked to indicate the frequency of symptoms on a 4-point scale (0=less than a day, 1=1 to 2 days, 2=3 to 4 days, 3=5 to 7 days). Positively formulated items were reversed. The total score consisted of the sum of all 20 items and ranged from 0 to 60 (Cronbach alpha=.88). A CES-D score ≥16 indicated the likelihood of a clinically significant depression [36].

All questionnaires above stem from validated and well-tested measurement tools in the Chinese version used in previous studies [25,26,35,36]. In addition, pretests were conducted to ensure the usability and technical functionality of the electronic versions of the questionnaires before the main study.

### Data Analysis

All data analyses were performed using IBM SPSS 24.0. The analyses of dropout and comparison of participant characteristics at T1 were conducted with independent sample *t* tests and chi-square tests. The 5% level (2-tailed) was used as the statistical significance cutoff point.

Intervention effects on PA and FVC behaviors (hypothesis 1a) were tested with repeated-measures analysis of variance (ANOVA). Hypothesis 1b on the combined healthy lifestyle indicator was tested using chi-square and logistic regression analysis (determining odds ratio, OR). In addition, intervention effects on social-cognitive variables (indicators of internal resources and external resources; hypothesis 2) and health outcomes (BMI, quality of life, and depression level; hypothesis 3) were evaluated by conducting a series of repeated-measures analyses of covariance (ANCOVA) with baseline behavior as the covariate. The 2 factors for both ANOVAs and ANCOVAs were time (T1 and T2) as the within-participants factor and treatment (IG vs WCG) as the between-participants factor. Hypothesis 4 on the multiple mediation models was performed using an SPSS macro [37]. Before the mediation analysis, the diagnostics of multicollinearity were performed. The criteria of serious multicollinearity problem include high correlation (*r*>.85), low tolerance (≤0.01), high variance inflation factor (VIF >10), low eigenvalue (approach zero), and large condition index (>30) among mediators [38]. Residualized change scores were used, and 95% CIs of the standardized effects of the intervention were estimated by applying the bootstrap approach (5000 bootstrap samples).

Results were reported based on those cardiac patients who participated in both measurement points. As less than 5% of items were missing and belonged to a randomized distribution, the expectation-maximization method was adopted to impute the missing data within each measurement point in time [39].

## Results

### Sample Characteristics and Randomization Check

There were 114 cardiac patients who participated in data collection at T1 (IG=60, WCG=54), and 83 of them completed data collection at T2 (IG=44, WCG=39). The dropout rate of participants was 27.2% (31/114) from T1 to T2. For participants in the IG, 44 (T2) out of 60 (T1) patients (73%) adhered to the whole 8-week intervention program. The 83 final patient sample included more females (57%, 48/83) than males, aged from 20 to 75 years (mean=49.18 years, SD=13.96). The majority of patients were in a relationship (94%, 78/83). Most patients had a high education level (89%, 74/83). The average BMI of patients was 23.65 (SD 2.89, range 16.71-31.25).

Participants at T1 and T2 did not significantly differ from dropouts at T2 regarding the gender (χ^2^_1_=2.3, *P*=.13), education level (χ^2^_1_=1.6, *P*=.20), relationship status (χ^2^_1_=0.3, *P*=.56), age (*t*_112_=−0.77, *P*=.44), amount of PA at T1 (*t*_109_=−1.78, *P*=.08), and FVC at T1 (*t*_108_=−0.88, *P*=.38).

Results also indicated that there were no significant differences across the 2 groups at T1 with respect to single behaviors of PA and FVC, internal resources for PA and FVC (combination of intention, self-efficacy, and planning), an external resource for PA and FVC (social support), age, or health outcomes (BMI, quality of life, and depression; all *P*=.11 to .86). In addition, there were no significant differences between the 2 groups on gender (χ^2^_1_=0.4, *P*=.54), education level (χ^2^_1_=0.8, *P*=.49), relationship status (χ^2^_1_=0.02, *P*=.90), and combined health lifestyle indicator (combination of both behaviors; χ^2^_1_=0.5, *P*=.50).

### Evaluation of Time and Treatment on Single-Behavior Indicators as well as Social-Cognitive Indicators of Behavior Change

**Figure 2:**
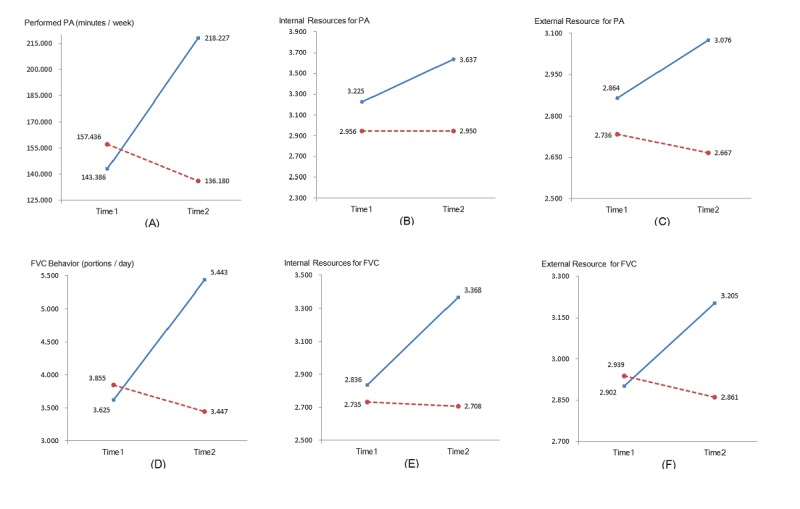
Mean values for intervention group (solid line) and waiting control group (dotted line) at T1 and T2. (A) Performed physical activity (PA) (minutes/week). (B) Internal resources for PA (combination of intention, self-efficacy, and planning). (C) External resources for PA (social-support). (D) Fruit and vegetable consumption (FVC) behaviour (portions/day). (E) Internal resources for FVC (combination of intention, self-efficacy, and planning). (F) External resources for FVC (social-support).

Table 1 presents the evaluation result of the time, treatment, and time x treatment effect. Out of 6 interaction effects, 5 were statistically significant, with an effect size of η^2^ranging from 0.06 to 0.43. The mean values of PA, FVC, internal resources of PA, internal resources of FVC, and social support of FVC between 2 groups over time are presented in Figure 2.

Although there was no significant interaction effect on social support for PA (*P=*.08), the mean value of social support for PA (see Figure 2, panel C) indicated that there was a descriptive difference between the 2 groups, which was clearly in favor of the IG.

### Evaluation of Time and Electronic Health Treatment on Combined Healthy Lifestyle Indicators

As the purpose of the intervention was not only to change single behaviors but also to improve patients’ lifestyle including both PA and FVC behaviors, change in the combined health indicator was evaluated. According to their PA and FVC behaviors, patients were categorized into groups based on whether or not they met the recommended criteria. The frequencies and percentage of a healthy and an unhealthy lifestyle at T2 depending on T1 lifestyle and treatment are presented in Table 2.

**Table 1:** Intervention efficacy evaluated in terms of changes over time assessed in a 2-factorial repeated measures analysis of variance.

Variables		Time				Treatment				Baseline behavior				Interaction time x treatment		
		*F* test (*df*)	η^2^		*P* value	*F* test (*df*)	η^2^	*P* value	*F* test (*df*)		η^2^	*P* value	*F* test (*df*)		η^2^	*P* value
**Physical activity (minutes/week)**																
	Behavior^a^	2.88 (1,81)		.03	.09	1.33 (1,81)	.02	.25	N/A^b^		N/A	N/A	9.25 (1,81)		.10	.003
	Internal resources^c^	14.13(1,80)		.15	<.001	11.17 (1,80)	.12	.001	13.66 (1,80)		.15	<.001	13.0 (1,80)		.14	.001
	External resource (social support)	3.26 (1,80)		.04	.08	2.51 (1,80)	.03	.12	0.19 (1,80)		<.01	.66	3.08 (1,80)		.04	.08
**Fruit and vegetable consumption (portions/day)**																
	Behavior	23.69 (1,80)		.23	<.001	7.27	.08	.009	N/A		N/A	N/A	59.01 (1,80)		.43	<.001
	Internal resources	11.06 (1,79)		.12	.001	9.21 (1,79)	.10	.003	10.21 (1,79)		.11	.002	24.96 (1,79)		.24	<.001
	External resource (social support)	1.59 (1,79)		.02	.21	1.05 (1,79)	.01	.30	0.46 (1,79)		<.01	.31	4.61 (1,79)		.06	.04

^a^Behavior indicators: moderate-vigorous physical activity or fruit and vegetable consumption.

^b^N/A: not applicable.

^c^Internal resources: intention, self-efficacy, and planning.

**Table 2:** Performance of a healthy and an unhealthy lifestyle at T2 based on lifestyle and treatment at T1.

Lifestyle at T1 and treatment		Lifestyle T2^a^, n (%)			Total, N	
			Unhealthy T2	Healthy T2		
**Unhealthy T1**						
	Intervention group		26 (72)	10 (28)		36
	Control group		33 (97)	1 (3)		34
**Healthy T1**						
	Intervention group		0 (0)	8 (100)		8
	Control group		2 (40)	3 (60)		5
**Unhealthy and Healthy T1**						
	Intervention group		26 (59)	18 (41)		44
	Control group		35 (90)	4 (10)		39
	Total		61 (74)	22 (27)		83

^a^Lifestyle T1 or T2=1 indicates meeting both behavior recommendations (conducting ≥150 min of moderate-vigorous physical activity per week and adopting ≥5 portions of fruit and vegetables per day); lifestyle T1 or T2=0 indicates not meeting both behavior recommendations (conducting ≥150 min of physical activity per week and adopting ≥5 portions of fruit and vegetables per day).

Descriptively, among those with an unhealthy lifestyle at T1, the IG outperformed the WCG in performing healthy lifestyle behavior (ie, 24.9% more). For those with a healthy lifestyle at T1, 100% (8/8) of patients in the IG still maintained their healthy lifestyle throughout treatment, compared with only 60% of patients in WCG. If all patients were considered together, it was found that 40% (18/44) of patients in the IG adopted a healthy lifestyle through treatment compared with only 10% of (4/39) patients in the WCG. However, due to the small sample size, the differences could not be statistically tested.

To further explore which variables predict healthy lifestyle behavior at T2, logistic regression analyses were employed with 3 models (Table 3). First, gender and age (all at T1) were used as predictors for the adoption of a healthy lifestyle at T2. Neither of these 2 variables, however, was a significant predictor (*P*=.30 to .47). Lifestyle at T1 was then added as a predictor in model 2, which exhibited a significant correlation to a healthy lifestyle at T2: patients who had a healthy lifestyle at T1 were about 47.51 times more likely to also adopt or maintain a healthy lifestyle at T2 (Table 3). Furthermore, the treatment was included as a predictor in model 3, which was also a significant predictor for a healthy lifestyle at T2 (*P* ≤.006; Table 3). In other words, those patients receiving the intervention were about 21.32 times more likely to practice or maintain a healthy lifestyle in comparison with patients in the control group. With model 3, lifestyle at T1 and treatment could attribute to 37% of the variance of a healthy lifestyle at T2.

### Evaluation of Time and Electronic Health Treatment on Health Outcomes

**Figure 3:**
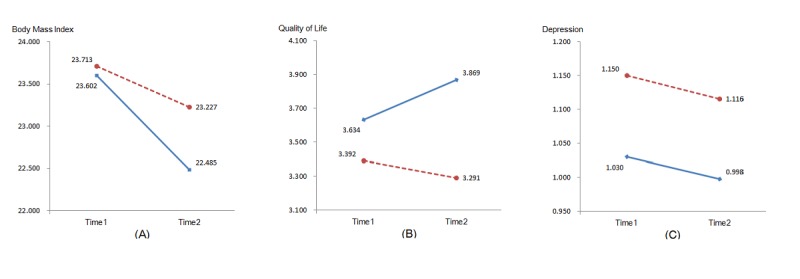
Mean values of health outcomes (Body mass index, quality of life, and depression) for intervention group (solid line) and waiting control group (dotted line) at T1 and T2. (A) Body mass index. (B) Quality of life. (C) Depression.

Analyses of the time and treatment effect on health outcomes in terms of BMI, quality of life, and depression level were performed. A significant difference was yielded for the interaction factor (time x treatment) on quality of life (*F*_1,79_=16.36, *P*<.001, η^2^=.17). Regarding the BMI and depression level, however, the interaction of time and treatment were not significant, respectively (F_1,78_=3.35, *P*=.07, η^2^=.04; *F*_1,80_=0.001, *P*=.98, η^2^<.001). The mean values for BMI, quality of life, and depression at T1 and T2 are displayed in Figure 3.

### Testing Mechanism of How the Electronic Health Treatment Facilitated a Healthy Lifestyle

**Figure 4:**
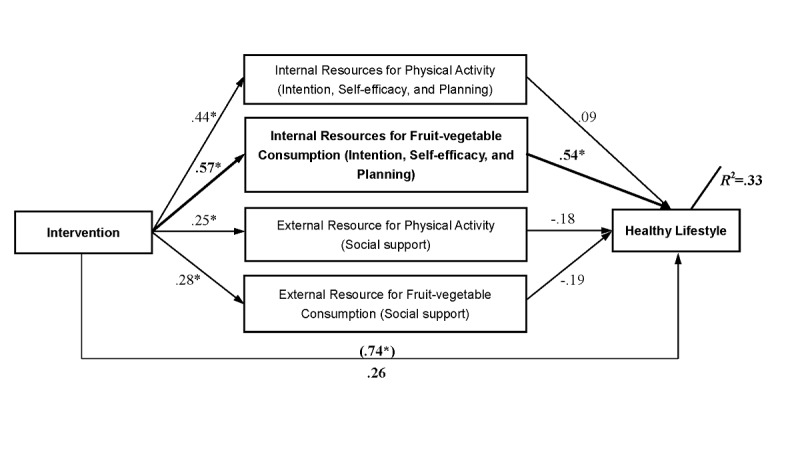
Mediation of the effect of the intervention on lifestyle changes by internal and external resources. Significant changes are indicted by an asterisk.

Finally, a multiple mediator analysis tested whether the effects of the intervention on lifestyle change could be explained by changes in internal and external resources (Figure 4). The multicollinearity diagnostics indicated that there was no serious multicollinearity problem among mediators (*r*=0.26-0.55, tolerance=0.48-0.60, VIF=1.66-2.07, eigenvalue=0.25-2.43, and condition index=1.00-3.11). Residualized change scores obtained by regression T2 scores on T1 scores were chosen for the proposed mediators (Figure 4). Group assignment predicted changes in all social-cognitive variables, in particular, in internal resources for PA (beta=.44, SE .81; *P*<.001), external resource for PA (beta=.25, SE .94; *P*=.02), internal resources for FVC (beta=.57, SE .68; *P*<.001, shown in bold in Figure 4), and external resource for FVC (beta=.28, SE .92; *P*=.009). Lifestyle change, as operationalized by meeting the recommendation toward PA and FVC, was predicted by changes in internal resources for FVC (beta=.54, SE .13; *P*<.001, shown in bold in Figure 4) and by no other variables. After controlling for changes in these predictor variables, the relation between group assignment and behavior change was no longer significant (beta=.26, SE .23; *P*=.27; without controlling: beta=.74, SE .21; *P*<.001), which indicates that internal resources for FVC was a full mediator of the intervention effectiveness. The multiple mediator model accounted for 33% of the variance (R^2^_adj_=.29; *P*=.001) in lifestyle.

**Table 3:** Predicting lifestyle at T2.

Variable	Model 1			Model 2			Model 3	
	OR (95% CI)	*P* value	OR (95% CI)		*P* value	OR (95% CI)		*P* value
Constant	0.71	N/A^a^	0.05		N/A	0.002		N/A
Gender	1.46 (0.53-4.04)	.47	2.60 (0.65-10.29)		.18	2.89 (0.65-12.79)		.16
Age	0.98 (0.95-1.02)	.30	1.01 (0.97-1.06)		.56	1.03 (0.98-1.08)		.30
Lifestyle in T1^b^	N/A		47.51 (6.97-324.02)		<.001	145.60 (11.24-1886)		<.001
Treatment^c^	N/A		N/A			21.32 (2.40-189.20)		.006
R^2^	.02	*<*.05	.27		<.001	.39		<.001
△R^2^	N/A	N/A	.25		<.001	.12		<.001

^a^N/A: not applicable.

^b^Lifestyle T1 or T2=1 indicates meeting both behavior recommendations (conducting ≥150 min of physical activity/week and adopting ≥5 portions of fruit and vegetables/day); lifestyle T1 or T2=0 indicates not meeting both behavior recommendations (conducting ≥150 min of moderate-vigorous physical activity/week and adopting ≥5 portions of fruit and vegetables/day).

^c^Treatment: 0 indicates waiting control condition; 1 indicates intervention condition.

## Discussion

### Principal Findings

The aim of this study was to gain insights into an 8-week Web-based health promotion intervention for Chinese cardiac rehabilitation patients after discharge from hospital. The majority of the research hypotheses were supported.

The main expected intervention effects on single behavior and combined lifestyle were identified. Compared with patients in the control group, patients in the IG reported significantly less decrease of PA and FVC over time (hypothesis 1a). The findings on multiple behavior change are in line with previous studies, which showed effects on both behaviors for patients with metabolic syndrome [40,41].

Furthermore, testing the effect of the intervention on lifestyle indicated that more patients in the IG than in the control group improved their lifestyle by meeting the recommendations for PA and FVC, with a 21 times higher likelihood to practice or maintain a healthy lifestyle in comparison with patients in the control group, after controlling for baseline behavior (hypothesis 1b). In addition, patients who already had a healthy lifestyle at baseline were 47 times more likely to engage in a healthy lifestyle than patients in the control group, after controlling for gender and age, which were both insignificant. The effect of baseline behavior matches previous findings. For instance, this was found in another study targeting employees at the workplace [20] and in rehabilitation settings in North America and Europe [15,33,42]. The findings of this study performed in China support the merits of a Web-based extended program for cardiac rehabilitation patients to improve their lifestyle and thus comparably improve their health, for the Western and the Eastern Hemisphere. Taking the different findings together, hypothesis 1a and hypothesis 1b were confirmed.

The hypothesized intervention effects on internal (combination of intention, self-efficacy, and planning) and external (social support) resources of behaviors were also identified—3 out of 4 social-cognitive test variables were found to be significant. The Web-based intervention increased the internal resources in the PA domain; it was even able to improve nutrition-related internal and external resources compared with the control group. The majority of hypothesis 2 was confirmed.

When testing the hypothesis on the intervention effects on health outcome, we found patients in the IG were more successful in improving their quality of life, which is coherent with the latest literature using a similar intervention program among university students [25]. However, the significant time x treatment interaction was not detected for BMI, although differential intervention effects surfaced on a descriptive level. In a systematic review on interactive computer-based interventions for weight loss or weight maintenance in overweight or obese people, it was indicated that treatment duration of such interventions significantly leading to weight loss is 6 months [42]. Likewise, the reduction of BMI through Web-based interventions in cardiac patients also needs long-term treatment duration in the future. In addition, we did not find the expected intervention effect on depression. The possible cause might be that the high percentage of cardiac rehabilitation patients (68%, 56/82) who reported their depression score at the start of the study was equal or higher than 16, which reflects the critical mental status of many patients. To reduce depression levels in cardiac patients, long-term and combined provision of guidance, information, stress management, and relaxation skills incorporated into the intervention would need to be developed in the future. This suggestion is also supported by another intervention study in rehabilitation patients with myocardial infarction [43]. Overall, hypothesis 3 was confirmed with one-third of the tested variables.

The hypothesized changes in internal and external resources of behavior change (combination of intention, self-efficacy, and planning; social support) were found in the majority of the tested variables in the mediation analysis. In addition, based on the sequential intervention mode in this study (first 4 weeks for PA, followed by latter 4 weeks for FVC), the gateway effect of the PA mechanism on nutrition was confirmed with the increasing close relationship from PA to nutrition on internal (*R*^2^from .44 to .57) and external (*R*^2^from .25 to .28) resources, which was also found before [23,44]. However, internal resource for FVC was the only facilitator of the intervention effectiveness. In particular, in the mediation model, it was found that patients in the IG who gained more internal resources on nutrition (eg, increased intention, higher self-efficacy, and more specific planning; Figure 2, panel E) were more likely to adopt or maintain a healthy lifestyle (Figure 4). Thus, it is important to empower the motivation to change, as well as the self-regulation ability of cardiac rehabilitation patients regarding nutrition behavior. This can boost patients to eat healthier and to maintain or adopt PA. Referring back to hypothesis 4, the data support the assumption of a mediator. However, only the internal resource of FVC was revealed to be a mediator but no internal resources of PA or external resource of PA and FVC. Thus, hypothesis 4 was only partially supported.

### Limitations

This study is subject to some limitations. First, as it was a small study, it provides only a limited sample size and power. Thus, the statistical analysis of chi-square could not be computed due to the small cell distributions. Second, all variables used in the study were self-report measures. Therefore, the inclusion of objective measures such as accelerometers, wearable cameras, or height and weight measurements is desirable in future studies. Third, physical health outcomes in the study only included BMI, which is too simple to reflect the comprehensive health situation of cardiac patients. More clinical health indicators such as blood pressure, total cholesterol, low-density lipoprotein cholesterol, high-density lipoprotein cholesterol, triglycerides, peak VO_2_, VO_2_@AT, and HR@AT should be added in the future. Fourth, only short-term effects were investigated. Long-term effects should be addressed in depth in the future with such an eHealth intervention. Fifth, patients received monetary incentives in exchange for their participation in the study, which might lead to a bias when evaluating the treatment effects. Moreover, the study could only include patients with access to a computer and the internet; therefore, the results cannot be generalized to patients without such access. Finally, although previously tested interventions in the Western Hemisphere [15,20] were tested with this study in the Eastern Hemisphere, no direct comparative study was conducted. It can only be concluded in this study that the intervention was as effective in Eastern Hemisphere as in Western Hemisphere. Future study should be warranted to test the same intervention in a comparative study to tease out differences in a systematic way.

### Conclusions

To conclude, the study demonstrated the potential of a Web-based multiple health behavior intervention among Chinese rehabilitation patients with CHD. The majority of study hypotheses were supported. Such an intervention enriches the extended rehabilitation approaches for cardiac patients to be active and remain healthy in daily life when they are discharged from the hospital. The mechanism of exploration indicates that internal psychological resource (eg, high intention and good self-regulation capability) is a central variable and should especially be considered in cardiac patient health promotion. In addition, gateway or transfer effects, from 1 behavior to another, should be addressed in depth to explore synergetic effects.

## Appendix

Multimedia Appendix 1 CONSORT‐EHEALTH checklist (V 1.6.1).

Multimedia Appendix 2 Intervention variables and content.

## Abbreviations

ANOVA: analysis of variance
ANCOVA: analyses of covariance
BMI: body mass index
CHD: coronary heart disease
CVD: cardiovascular disease
eHealth: electronic health
FVC: fruit and vegetable consumption
HAPA: Health Action Process Approach
IG: intervention group
PA: physical activity
OR: odds ratio
VIF: variance inflation factor
WCG: waiting control group
