# Supplementary figures and images for: Evaluation of a Web-Based Intervention for Multiple Health Behavior Changes in Patients With Coronary Heart Disease in Home-Based Rehabilitation: Pilot Randomized Controlled Trial
Source: J Med Internet Res. 2018 Nov 19;20(11):e12052. doi: 10.2196/12052 (PMC6277829; doi:10.2196/12052)

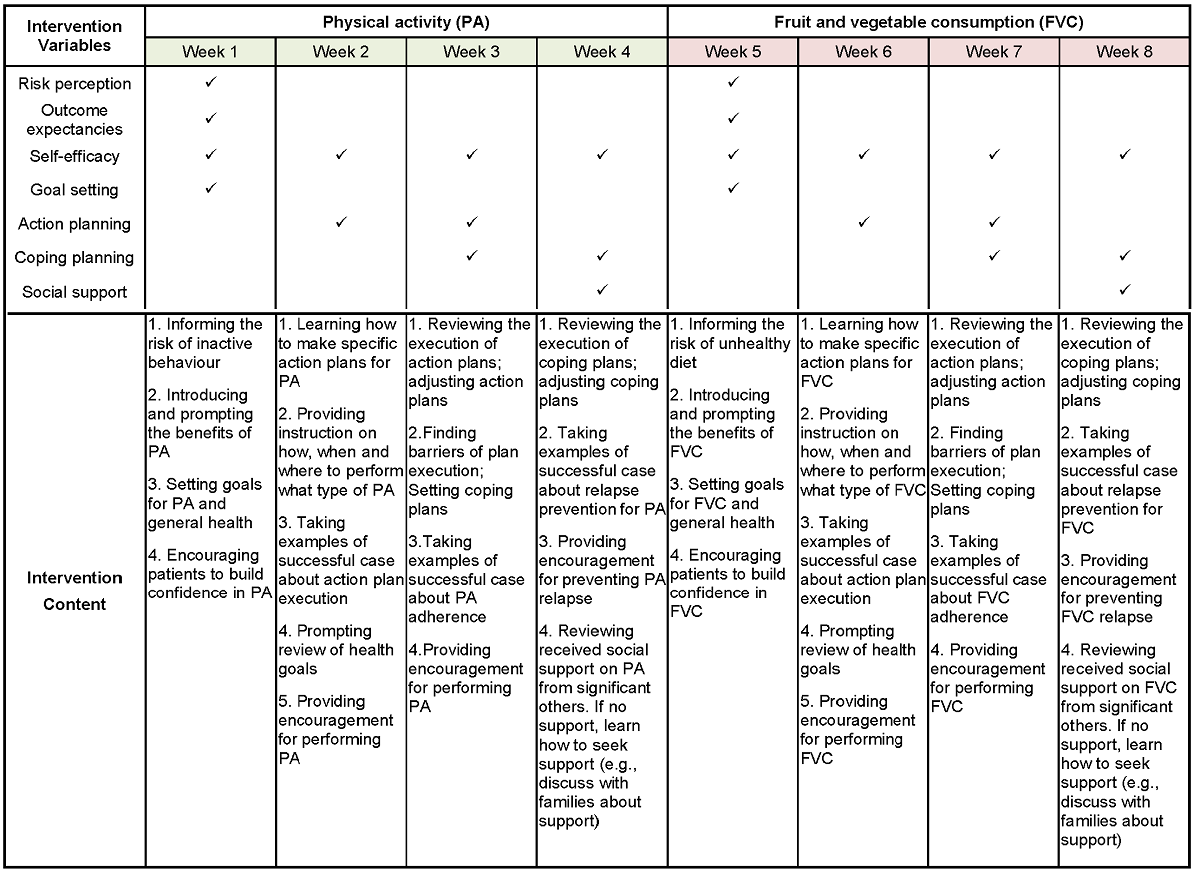

Supplement: Multimedia Appendix 2 [file jmir_v20i11e12052_app2.png]
